# Generalisable machine learning models trained on heart rate variability data to predict mental fatigue

**DOI:** 10.1038/s41598-022-24415-y

**Published:** 2022-11-21

**Authors:** András Matuz, Dimitri van der Linden, Gergely Darnai, Árpád Csathó

**Affiliations:** 1 Department of Behavioural Sciences, Medical School, University of Pécs, Szigeti Str. 12, Pécs, 7624 Hungary; 2 Department of Psychology, Education, and Child Studies, Erasmus University Rotterdam, Rotterdam, The Netherlands; 3 Department of Neurology, Medical School, University of Pécs, Pécs, Hungary; 4 MTA-PTE Clinical Neuroscience MR Research Group, Pécs, Hungary

**Keywords:** Human behaviour, Cognitive neuroscience, Computational biology and bioinformatics, Autonomic nervous system

## Abstract

A prolonged period of cognitive performance often leads to mental fatigue, a psychobiological state that increases the risk of injury and accidents. Previous studies have trained machine learning algorithms on Heart Rate Variability (HRV) data to detect fatigue in order to prevent its consequences. However, the results of these studies cannot be generalised because of various methodological issues including the use of only one type of cognitive task to induce fatigue which makes any predictions task-specific. In this study, we combined the datasets of three experiments each of which applied different cognitive tasks for fatigue induction and trained algorithms that detect fatigue and predict its severity. We also tested different time window lengths and compared algorithms trained on resting and task related data. We found that classification performance was best when the support vector classifier was trained on task related HRV calculated for a 5-min time window (AUC = 0.843, accuracy = 0.761). For the prediction of fatigue severity, CatBoost regression showed the best performance when trained on 3-min HRV data and self-reported measures (R^2^ = 0.248, RMSE = 17.058). These results indicate that both the detection and prediction of fatigue based on HRV are effective when machine learning models are trained on heterogeneous, multi-task datasets.

## Introduction

Mental fatigue (henceforward, *fatigue*) is a psychobiological state caused by engaging in cognitively demanding activities for extended period^1,2^. The literature shows that the main characteristic of fatigue is the feeling of resistance against further (cognitive) effort, which is sometimes—but certainly not always—followed by a decline in performance^2–4^. In general, fatigue is considered a multifaceted state that involves complex interactions between brain activity (e.g., norepinephrine and dopaminergic systems)^5–8^, subjective feelings (e.g., lack of energy) and cognitive performance^3,9,10^. Because fatigue tends to influence cognitive performance, it is known to be a topic that is highly relevant to human safety. For example, the risk of work-related and road accidents is substantially higher when people are fatigued^11,12^. Accordingly, the prevention as well as the detection of fatigue is imperative^13^. In line with this, research on the biomarkers of fatigue has become increasingly important and suggests that fatigue can be estimated on the basis of markers that reflect the activity of the cerebral cortex (e.g., theta activity obtained by means of neuroimaging techniques) or the autonomous nervous system (e.g. Heart Rate Variability [HRV]) to prevent its negative consequences^14^.

Machine learning is a relatively novel way of utilising biomarkers to detect fatigue and this approach has captured the attention of science and practice alike^15–18^. A few studies have used biological signals obtained with electroencephalography (EEG) to train machine learning models that are capable of effectively detecting mental fatigue (i.e., classification models that successfully distinguish between fatigued and non-fatigued states; for a review, see Ref.^19^). Even though the high accuracy (> 80–90%) of these models is impressive, EEG has several limitations, such as the difficult and time-consuming procedure of setting up the electrodes and its sensitivity to external electromagnetic fields^20^. Because of these limitations, and the fact that fatigue has also been associated with changes in the autonomic nervous system^21^, other studies have investigated whether fatigue detection is possible based on biological signals obtained by peripheral measures, for example, electrooculography^22^ or electrocardiography (ECG)^23^. Most of the fatigue studies that have used ECG calculated the variation of consecutive R-wave peaks denoted in the literature as HRV^24^. HRV reflects the activity of the autonomic nervous system and is indeed a potential reliable biomarker of fatigue because many previous studies have confirmed the association between HRV and fatigue^21,25–28^.

Machine learning studies have shown that models trained exclusively on HRV are capable of effectively detecting fatigue with the best accuracy scores ranging between 75 and 91%^23,29–31^. Laurent et al. (2013), for example, showed that the support vector machine (SVM) algorithm trained on HRV data recorded during the performance of a fatiguing switching task (i.e., algorithm trained on task related HRV data) was able to detect fatigue with an accuracy up to approximately 80%. In contrast to this study, which used task related data, another study tested the algorithms based on resting HRV data^30^. In this study, the resting period preceding prolonged task performance was labelled the *non-fatigue state*, and the resting period after task performance was labelled the *fatigue state*. The authors found that the k-nearest neighbors (KNN) algorithm was capable of detecting fatigue with an accuracy of approximately 75% based on four HRV indices.

The differences in predictive accuracy reported in these studies was probably caused by various factors. The most important factors might be the methodological differences across the studies such as variations in the cognitive tasks used to induce fatigue, the time window used for HRV calculation, sample size differences (*N*s ranged between 13 and 45) or even in whether the ECG was recorded during rest or active task performance. In contrast to time window and sample size factors, the effects of which have been extensively studied in the machine learning literature^32,33^, no studies so far have used multiple cognitive tasks to induce fatigue or have directly compared the predictive performance of models trained on resting and task related HRV data even though this would have significant implications for both practice and research. Therefore, in the present study, we extend the literature in this field by analysing a multi-task dataset and comparing models trained on task related as well as resting HRV data.

In addition to predictive performance, the models are also supposed to demonstrate comprehensive generalisability, that is, to make accurate predictions on previously unseen samples^34,35^. To increase the generalisability of machine learning models, it is best using a reasonably large dataset, avoid information leakage (e.g. by performing feature selection only on the training dataset), conduct cross-validation and test the models on previously unseen data^36,37^. In addition, one might argue that using more than one cognitive task to induce fatigue might strengthen the generalisability because the different tasks affect different cognitive and affective systems^38^ and the models trained on such heterogeneous data may be less sensitive to noise and task-specific characteristics^39^. In other words, models that accurately detect fatigue irrespective of the type of task at hand are considered to be of greater value because they could be effectively utilised in a variety of situations, which reflects both higher reliability and more usefulness in practice.

In this study, we aimed to train machine learning models to achieve comprehensive generalisability and to become robust to task-specific characteristics. To achieve this goal, we first combined the datasets of three fatigue-related experiments that applied different cognitive tasks requiring different cognitive operations. Thus, the fatigue induction was variable. Second, for the same reason, we were able to conduct analyses on a relatively larger dataset (*n* = 85) than previous studies (highest *n* = 45)^29^. Third, to avoid information leakage, the data were pre-processed after the separation of training and test sets. Fourth, we trained the models using cross-validation on the training data set, but the final evaluation was based on a previously unseen data set so we could observe how well the models generalise to new data. This analytic approach differs from that of the previous studies^23,29–31^, which used cross-validation only and did not test the models on unseen holdout data.

In addition to fatigue detection, the prediction of the level of fatigue caused by prolonged cognitive performance is an important question that can be addressed by machine learning. In line with this, a few attempts have been made to train machine learning models on biomarkers and other variables such as demographics or psychometric data to predict the level of fatigue caused by engaging in cognitively demanding tasks^40–42^. Highly relevant to our study is Mun and Geng’s^65^, which used machine learning to predict the level of post-experiment subjective fatigue based on various types of self-reported and biological data including resting HRV. The most predictive features were self-reported measures (e.g., pre-experiment fatigue, anxiety) but other indices reflecting cardiac activity such as blood pressure and the low frequency HRV component also contributed to the prediction of post-experiment fatigue. Similar to studies that have used machine learning to detect fatigue, Mun and Geng induced fatigue by means of a single task. Thus, the question of whether post-experiment fatigue induced by different cognitive tasks can be predicted by models trained on pre-experiment variables remains unanswered.

In sum, the present study had three main goals. First, we trained classification algorithms to detect fatigue and regression algorithms to predict the severity of post-experiment subjective fatigue induced by prolonged cognitive performance based on a heterogeneous data set in terms of fatigue induction. Second, we compared the predictive performance of classification models trained on resting and task related HRV data. To our knowledge, no previous studies have made such a comparison. Third and last, we explored the effects of time window length to find the shortest time windows that still result in accurate predictions because the use of shorter ECG recordings would be beneficial for research as well as practice.

## Materials and methods

### Data base

For the analysis, we combined the datasets of three fatigue experiments that used similar procedures. The final dataset consisted of data from 85 healthy university students (33 males; *M* age = 21.71 years, *SD* = 2.53). All the participants provided written informed consent and the experiments were approved by the local ethical committee (nr. 7698). All experiments were carried out in accordance with the Code of Ethics of the World Medical Association (Declaration of Helsinki). Part of the findings of the first fatigue experiment were published in a previous article^26^, but the original dataset (*n* = 23) was extended by adding the data of 15 more participants, which thus far have not been published. Part of the findings of the second experiment (*n* = 20) were also published^27^. It is important to note that these previous studies had different goals and completely different analyses compared with the present study. The findings of the third experiment (*n* = 27) have not been published before.

### Fatigue induction

**Figure 1:**
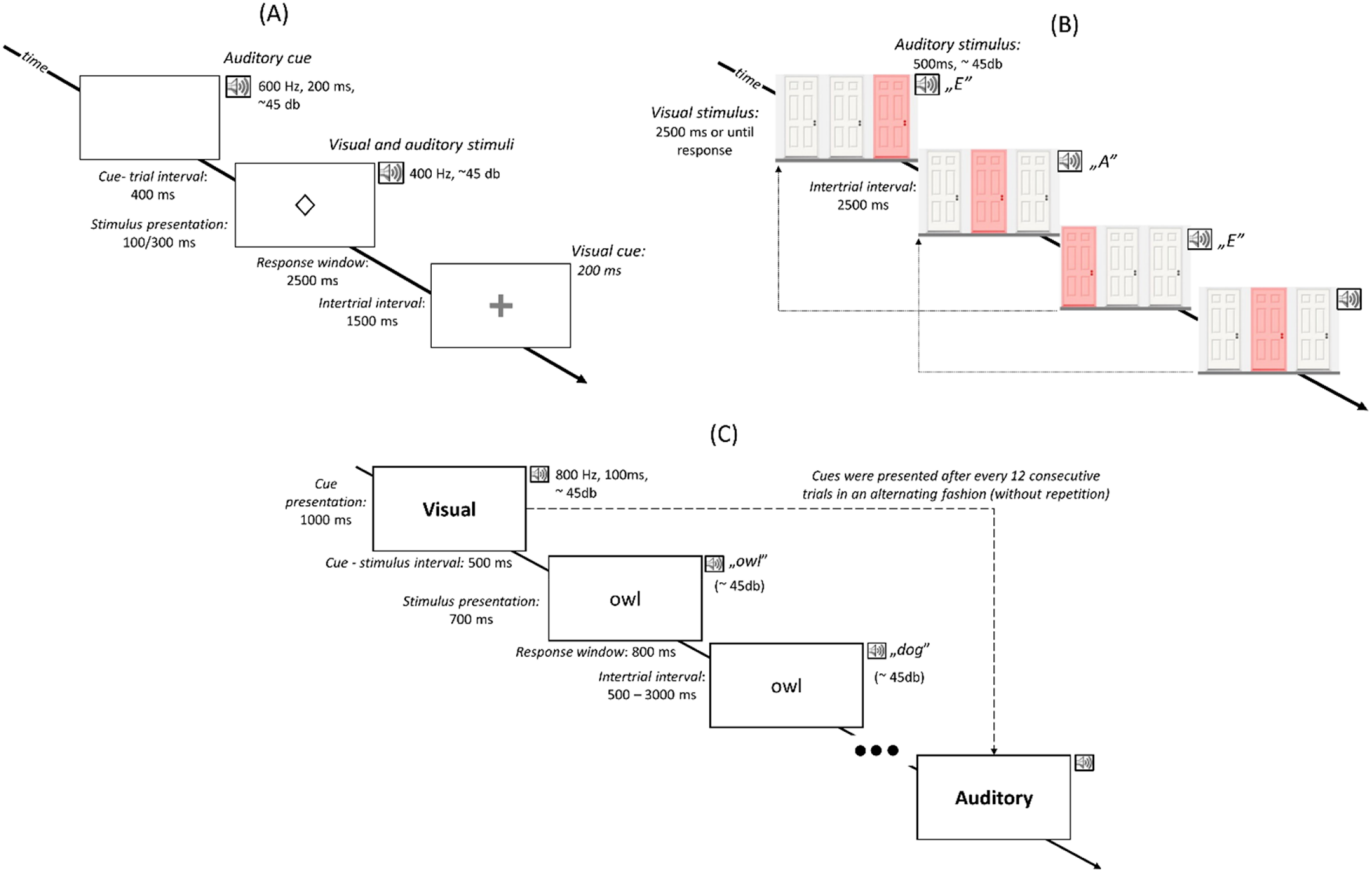
Schematised sequence of trials in the experiments. (**A**) In the task switching experiment, participants were required to decide the duration of the stimulus in the cued modality, while they had to ignore the stimulus presented in the other modality. (**B**) In the 2-back experiment, participants were asked to compare the auditory and visual stimuli in the actual trial to the stimuli presented two trials earlier and decide whether any of the two stimuli was identical to the one presented two trials earlier. (**C**) The Stroop experiment required participants to decide whether the word presented in the cued modality belonged to the semantic category of mammals or birds, while the word presented in the other modality had to be ignored.

Figure 1 schematises the sequence of trials in each experiment. In the first experiment (i.e., the task switching experiment), fatigue induction was achieved by a modality-switching task^26,43^, that required participants to make temporal judgements about either an auditory or a visual stimulus. In the second experiment (i.e., 2-back experiment)^27^, we used a bimodal 2-back task with a game-like character that presents auditory and visual stimuli simultaneously; participants were required to compare the actual stimuli with the ones presented two trials earlier^44^. Finally, the task used in the third experiment (i.e., the Stroop experiment) was a bimodal Stroop task that required participants to make sematic categorisations of written or spoken words. The tasks lasted approx. 1.2–1.5 h. For a detailed description of the tasks used for fatigue induction, see the Supplementary Materials.

Before performing the prolonged task, participants practiced the task at hand and a few subjective measurements were administered (see below). ECG signals were recorded (with three chest electrodes; Lead II.) for 5 min before (*pre-task resting*) and after (*post-task resting*) the task and were continuously recorded during the whole course of the task. In terms of the duration of resting ECG recording, however, the 2-back experiment differed from the other two experiments, because both the pre-task-, and post-task resting periods lasted only 4 min.

### Subjective measures

Subjective fatigue was assessed with visual analogue scales (VAS). Participants indicated their actual experience of fatigue on a 100-mm horizontal line with “*No fatigue at all*” and “*Very severe fatigue*” printed on the left and right end of the line, respectively. The VAS was administered two times: before and after the prolonged task performance. To further characterise the tasks used in the experiments, we applied the NASA Task Load Inventory (NASA_TLX_)^45^. The NASA_TLX_ is a self-reported measure that assess different aspects of perceived workload. It consists of six subscales with 21 gradations each: mental demand, physical demand, temporal demand, overall performance level, effort, and frustration level.

### Heart rate variability analysis

ECG data were sampled at a rate of 1 kHz by using a CED 1401 Micro II analogue–digital converter device (Cambridge Electronic Design, Cambridge, UK). After we obtained the ECG signal, it was further processed in Spike2 software (Cambridge Electronic Design, Cambridge, UK). First, the data were visually inspected, and artifacts were removed. Then, the inter-beat-intervals (i.e., RR-intervals) were extracted and analysed further with Kubios HRV analysis software^46^. The “very low” option for artifact correction was chosen and ectopic intervals were replaced using cubic spline interpolation. Time-domain, frequency-domain (using the Fast Fourier Transform routine), and non-linear analyses were applied to quantify HRV. In the time domain, we extracted the mean and the standard deviation of normal-to-normal NN intervals (mean RR and SDNN, respectively), minimum and maximum heart rate, root mean square of successive differences (RMSSD), the percentage of successive RR intervals that have a difference of more than 50 ms (pNN50) and the triangular index. The frequency-domain variables included the natural logarithms of the total power (TP) and the main frequency components such as very low frequency (VLF; 0–0.04 Hz), low frequency (LF; 0.04–0.15 Hz) and high frequency (HF; 0.15–0.4 Hz). The ratio of LF and HF (LF/HF) was also calculated. Finally, the obtained non-linear indices were the width (SD1) and length (SD2) of the Poincaré plot; their ratio (SD1/SD2); approximate entropy; sample entropy; correlation dimension (D2) and the detrended fluctuation analysis measures, DFA1 and DFA2.

In sum, we used 20 HRV indices for the analysis. These indices were calculated separately for the two resting periods (pre-task and post-task resting) and for two task-related periods (the beginning and the end of the task). To investigate the effects of time window length on the performance of the machine learning models, we calculated the HRV indices for different time windows ranging from 1 to 5 min.

### Classification algorithms

All programming was implemented in Python using the scikit-learn (Version 0.23.2.)^47^ and CatBoost (CB, Version 1.1.) packages^48^. For detailed mathematical background applied for model building, see the following studies^48–53^. The aim of classification was to develop models that are able to accurately distinguish between fatigue and non-fatigue states. We addressed this binary classification problem by training algorithms on resting HRV data and task related HRV data separately. For the algorithms trained on resting data, the pre-experiment resting HRV data were labelled the “*non-fatigue*” state and the post-experiment resting HRV data were labelled the “*fatigue*” state. For the algorithms trained on task-related data, the beginning period (i.e., the first 1–5 min depending on the time window) of task performance was labelled the “Non-fatigue” state, and the end of the task (i.e., the last 1–5 min depending on the time window) was labelled the “Fatigue” state. Fatigue was operationalised as an increase in subjective fatigue (measured by VAS) (for similar operationalizations, see e.g. Refs.^8,21,54–56^). Hence, the data of three participants who did not have a higher post-experiment subjective fatigue score compared with their pre-experiment score were excluded from the classification analyses. Thus, for the classification problems, the sample size was reduced to 82 participants. For each person, HRV was calculated for two intervals (one with the “*fatigue*” label and another with the “*non-fatigue*” label). The final data set that the algorithms used for each classification problem consisted of 164 data points for each HRV variable.

The data set was split into training (~ 70%) and test sets (~ 30%). Feature selection via recursive feature elimination with five-fold cross-validation (5-CV) was performed on the training set. Four classification algorithms were used: SVM, KNN, random forest, and CB classifier. Before the training, the data were standardised by z-transformation (except for the training of random forest and CB classifier). Hyperparameters for each classifier were optimised through grid search with 5-CV (for an elaborated description of hyperparameters tuning and feature selection, see the Supplementary Materials). Both the internal validation and the evaluation of the classification performance on the test set were done using the area under the receiver operating characteristic curve (area under the curve [AUC]). We also calculated accuracy, sensitivity and specificity were to evaluate the performance indicated by the testing data set. To test whether the fatigue detection performances were higher than chance level, permutation tests with 1000 iterations were carried out^57^ (for further details, see the Supplementary materials).

### Regression models

Similar to the classification problems, we carried out regression modelling with the scikit-learn^47^ and CB packages^48^. Because the data set used for regression modelling was smaller (because only the pre-experiment resting HRV was used), the ratio used for dividing the dataset into training and testing sets was 80%/20% so we would have a sufficiently large dataset for training. In the regression models, the outcome measure was the level of subjective fatigue after prolonged task performance measured on a VAS. Potential predictor variables included the 20 pre-experiment resting HRV indices, participants’ sex, age, self-reported sleep duration, pre-experiment subjective fatigue and task duration. We used the least absolute shrinkage and selection operator (LASSO) method to select the most important features^53^. As with the classification models, feature selection was performed separately for each time window.

LASSO and elastic net regression as well as the CB regressor algorithm were applied. The hyperparameter alpha was tuned for the LASSO and elastic net models, while the number of estimators, depth and the l2 leaf regularisation parameter were tuned for the CB regressor on the training set with 5-CV. The data used for LASSO and elastic net models were standardised with z-transformation. After parameter optimisation, the models predicted the level of subjective fatigue in the previously unseen testing dataset. To evaluate the performance, two metrics, the root mean square error (RMSE) and the *R*^2^, were calculated. Permutation tests with 1,000 iterations were carried out to assess the significance of the *R*^2^ values.

Finally, to reduce the effect of statistical fluctuations and potential biases due to the random splitting of the data, the entire procedure (i.e., random allocation of participants into training and test sets, feature selection, model training, parameter tuning, and evaluation) was repeated 100 times with different randomisation seeds for each of the classification and regression problems. The means and standard deviations of all evaluation metrics across the 100 iterations were used to assess the predictive performance and to obtain the 95% confidence interval (CI). However, for regression, the distributions of model performances were found to be skewed and therefore, we used the median to describe the centre of the distribution, and the Q1 and Q3 to describe the dispersion of the distribution. In addition, to help the interpretability of the results and to support the potential practical use, we also inspected the most average models (i.e., the ones with an AUC/*R*^2^ score closest to the mean) to gain insight into the optimal parameters.

### Ethical approval

The procedure of the experiments were reviewed and approved by the Ethics Committee of the Medical School, University of Pécs (nr. 7698).

## Results

### Fatigue induction and workload

In each experiment, subjective fatigue was significantly higher after the experiment than before (task-switching experiment: t(37) = 9.34, *p* < 0.001, *M*_diff_ = 32.41; 2-back experiment: t(19) = 6.81, *p* < 0.001, *M*_diff_ = 25.4; Stroop experiment 3: t(26) = 8.27, *p* < 0.001, *M*_diff_ = 27.37). This indicates that the fatigue manipulation was successful regardless of the cognitive task the participants performed. A univariate analysis of variance showed that the three experiments did not significantly differ in subjective fatigue before the task started (F(2,82) = 2.605, *p* = 0.08, η_*p*_^2^ = 0.06). The analysis of NASA_TLX_ scores, however, revealed that the tasks in the three experiments differed in terms of perceived workload. Univariate analyses of variance yielded significant Experiment main effects for mental demand (F(2,82) = 4.247, *p* < 0.05, η_*p*_^2^ = 0.09), physical demand (F(2,82) = 3.471, *p* < 0.05, η_*p*_^2^ = 0.08), temporal demand (F(2,82) = 3.482, *p* < 0.05, η_*p*_^2^ = 0.08), effort (F(2,82) = 5.991, *p* < 0.01, η_*p*_^2^ = 0.13), and frustration (F(2,82) = 3.836, *p* < 0.05, η_*p*_^2^ = 0.09). Bonferroni-corrected pairwise comparisons showed that the Stroop task was perceived to be less mentally demanding (*M* = 14.63, *SD* = 3.91) and required a lower level of effort (*M* = 12.96, *SD* = 3.78) than the switching task (mental demand: *M* = 16.71, *SD* = 3.35; effort: *M* = 15.87, *SD* = 3.96) and the 2-back task (mental demand: *M* = 17.10, *SD* = 2.02; effort: *M* = 15.16, *SD* = 3.18). The Stroop task was also perceived to be less temporally demanding (*M* = 9.59, *SD* = 4.92) and less frustrating (*M* = 7.96, *SD* = 5.42) compared with the switching task (temporal demand: *M* = 12.53, *SD* = 4.64; frustration: *M* = 11.63, *SD* = 5.84). All corrected *p*-values were < 0.05.

### Classification

**Figure 2:**
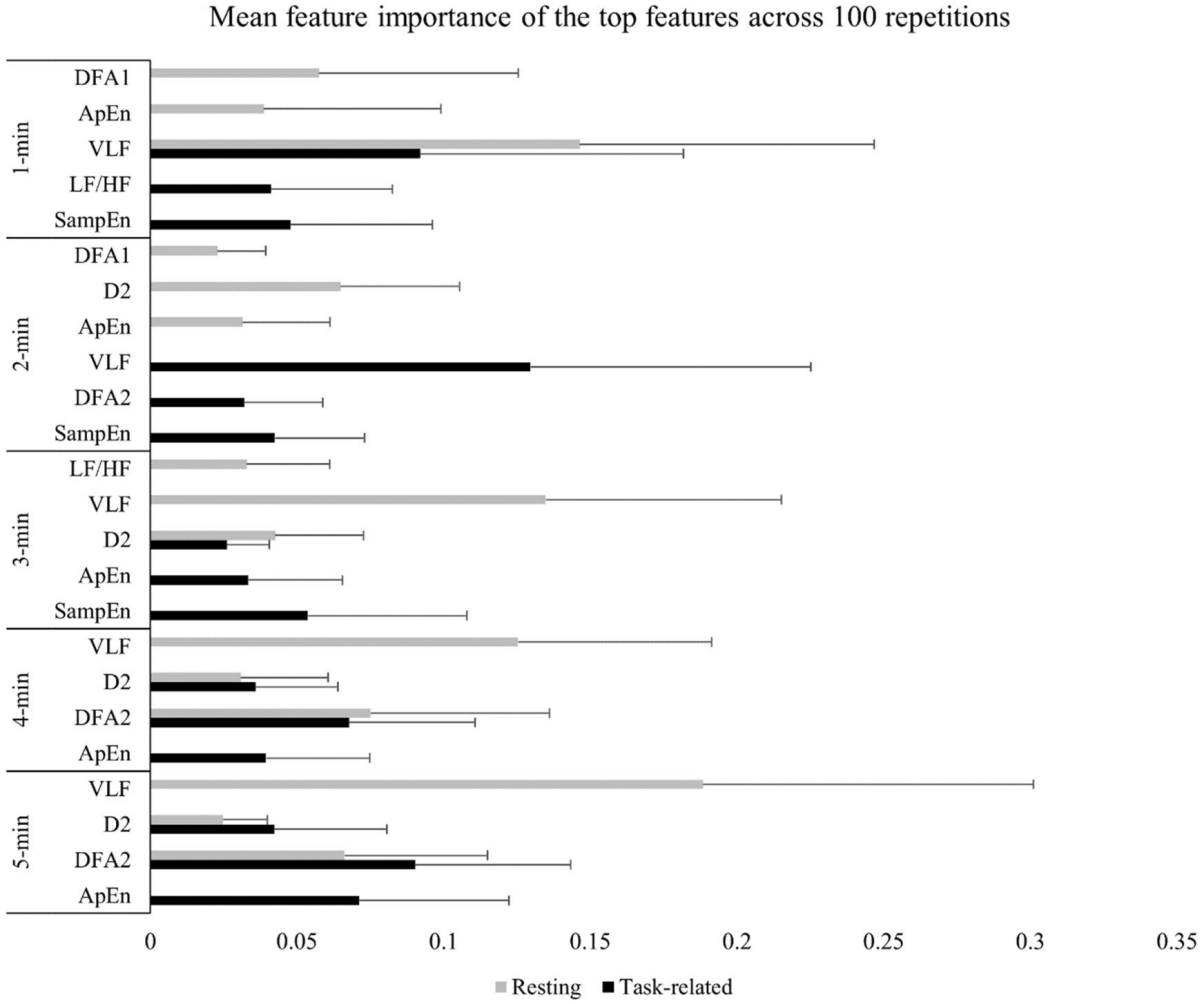
The top 3 most frequently selected features for classification. Error bars represent 1 standard deviation. *ApEn* approximate entropy, *D2* correlation dimension, *DFA1* short-term fluctuation obtained by detrended fluctuation analysis, *DFA2* long-term fluctuation obtained by detrended fluctuation analysis, *LF/HF* ratio of the low and high-frequency components, *SampEn* sample entropy, *VLF* natural logarithm of the very low frequency component.

**Figure 3:**
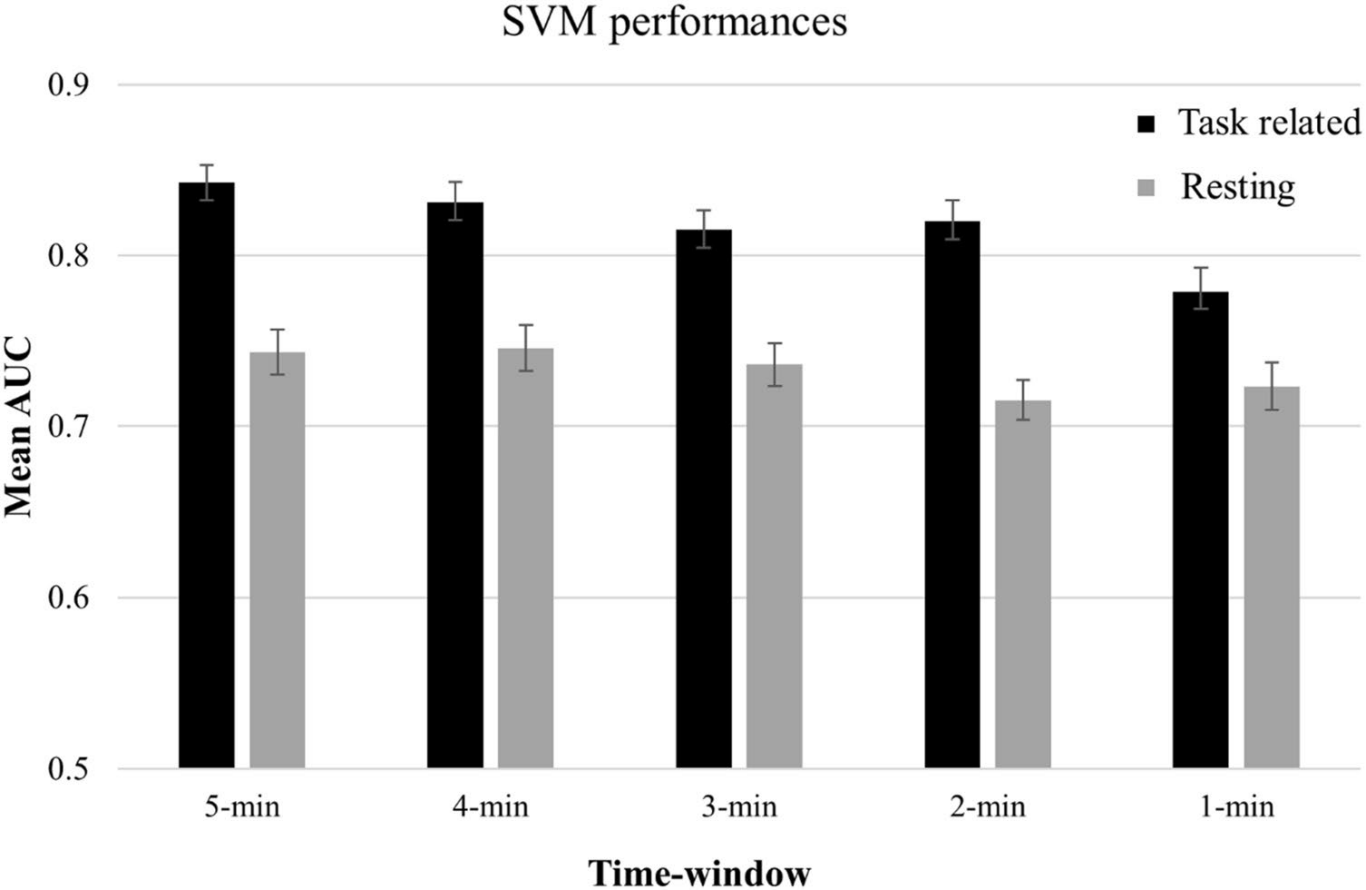
Performances of the support vector classifiers in each classification problem. Error bars represent the 95% confidence intervals *AUC* area under the receiver operating characteristic curve, *SVM* support vector machine.

Using the HRV data, we trained classification models to successfully differentiate fatigue and non-fatigue states. Figure 2 presents the most important features for each classification problem. The results of cross-validation in the training set are reported in the Supplementary Materials (see Supplementary Table S1). Predictive performance of the classifiers trained on task related and resting HRV data for the test set are summarised in Tables 1 and 2, respectively. Below, we report the most important findings. Permutation tests demonstrated that the classification performance of all classifiers in all classification problems differed significantly from the permutated null distributions. The performance of the SVM, KNN, RF, and CB classifiers produced similar results in each classification problem. However, on average across all classification problems, the SVM slightly outperformed the other three classifiers. Irrespective of the classifier and time window used, we observed that, compared with the resting HRV data, the algorithms were more accurate in predicting fatigue states in the task related HRV data (see Fig. 3). In addition, as expected, the length of the time window had an effect on model performance because in most of the cases the training on longer time windows resulted in better performances compared with shorter time windows.

**Table 1:** Results of the classifiers trained on task related HRV.

Time window/algorithm		Evaluation metrics (test set)				
		Mean AUC (95% CI)	Mean accuracy (95% CI)	Mean sensitivity (95% CI)	Mean specificity (95% CI)	*p*
5-min	SVM	0.843 (0.833–0.853)	0.761 (0.750–0.772)	0.726 (0.712–0.740)	0.795 (0.782–0.809)	0.009
	KNN	0.821 (0.810–0.832)	0.746 (0.735–0.757)	0.706 (0.690–0.721)	0.787 (0.771–0.802)	0.004
	RF	0.813 (0.803–0.823)	0.733 (0.724–0.742)	0.741 (0.726–0.755)	0.725 (0.708–0.742)	0.003
	CB	0.812 (0.800–0.824)	0.735 (0.723–0.747)	0.748 (0.730–0.766)	0.723 (0.706–0.740)	0.010
4-min	SVM	0.831 (0.819–0.843)	0.743 (0.730–0.756)	0.702 (0.687–0.716)	0.785 (0.768–0.801)	0.013
	KNN	0.811 (0.800–0.822)	0.737 (0.726–0.748)	0.666 (0.651–0.682)	0.807 (0.793–0.821)	0.005
	RF	0.811 (0.800–0.822)	0.734 (0.722–0.746)	0.720 (0.702–0.739)	0.748 (0.733–0.763)	0.006
	CB	0.807 (0.796–0.818)	0.728 (0.716–0.74)	0.708 (0.689–0.727)	0.749 (0.733–0.765)	0.009
3-min	SVM	0.815 (0.803–0.827)	0.735 (0.723–0.747)	0.697 (0.683–0.711)	0.772 (0.756—0.788)	0.015
	KNN	0.786 (0.774–0.798)	0.718 (0.706–0.730)	0.646 (0.629–0.664)	0.789 (0.773–0.805)	0.006
	RF	0.781 (0.770–0.792)	0.716 (0.705–0.727)	0.706 (0.688–0.724)	0.726 (0.708–0.743)	0.006
	CB	0.793 (0.782–0.804)	0.726 (0.715–0.737)	0.708 (0.689–0.727)	0.744 (0.728–0.760)	0.015
2-min	SVM	0.820 (0.808–0.832)	0.744 (0.731–0.757)	0.725 (0.710–0.740)	0.763 (0.748–0.778)	0.005
	KNN	0.808 (0.796–0.820)	0.736 (0.724–0.748)	0.685 (0.668–0.702)	0.787 (0.773–0.802)	0.005
	RF	0.792 (0.780–0.804)	0.722 (0.709–0.735)	0.726 (0.708–0.744)	0.718 (0.697–0.739)	0.007
	CB	0.800 (0.789–0.811)	0.732 (0.720–0.744)	0.725 (0.707–0.743)	0.739 (0.722–0.756)	0.007
1-min	SVM	0.779 (0.765–0.793)	0.717 (0.703–0.731)	0.680 (0.664–0.696)	0.754 (0.736–0.771)	0.018
	KNN	0.764 (0.751–0.777)	0.712 (0.700–0.724)	0.656 (0.638–0.674)	0.768 (0.753–0.783)	0.009
	RF	0.763 (0.751–0.775)	0.698 (0.686–0.710)	0.690 (0.671–0.709)	0.706 (0.688–0.725)	0.007
	CB	0.760 (0.747–0.773)	0.698 (0.686–0.710)	0.675 (0.658–0.692)	0.721 (0.701–0.741)	0.015

*AUC* area under the receiver operating characteristic curve, *CB* CatBoost classifier, *CI* confidence interval, *KNN* k-nearest neighbors, *p* permutation test p-value, *RF* random forest, *SVM* support vector machine.

**Table 2:** Results of the classifiers trained on resting HRV.

Time window/algorithm		Evaluation metrics (test set)				
		Mean AUC (95% CI)	Mean accuracy (95% CI)	Mean sensitivity (95% CI)	Mean specificity (95% CI)	*p*
5-min	SVM	0.743 (0.730–0.756)	0.701 (0.689–0.713)	0.709 (0.692–0.726)	0.693 (0.678–0.709)	0.035
	KNN	0.738 (0.725–0.751)	0.701 (0.689–0.713)	0.671 (0.650–0.691)	0.731 (0.712–0.750)	0.026
	RF	0.731 (0.716–0.746)	0.691 (0.678–0.704)	0.685 (0.663–0.706)	0.696 (0.678–0.715)	0.027
	CB	0.731 (0.717–0.745)	0.689 (0.676–0.702)	0.686 (0.665–0.707)	0.693 (0.673–0.713)	0.040
4-min	SVM	0.746 (0.732–0.760)	0.699 (0.686–0.712)	0.719 (0.702–0.736)	0.678 (0.660–0.696)	0.014
	KNN	0.750 (0.738–0.762)	0.702 (0.690–0.714)	0.653 (0.634–0.672)	0.751 (0.735–0.768)	0.003
	RF	0.730 (0.717–0.743)	0.678 (0.665–0.691)	0.657 (0.639–0.675)	0.698 (0.680–0.717)	0.013
	CB	0.733 (0.719–0.747)	0.686 (0.673–0.699)	0.659 (0.641–0.677)	0.712 (0.695–0.729)	0.014
3-min	SVM	0.736 (0.723–0.749)	0.683 (0.670–0.696)	0.665 (0.649–0.682)	0.702 (0.684–0.719)	0.023
	KNN	0.738 (0.725–0.751)	0.686 (0.675–0.697)	0.604 (0.587–0.621)	0.768 (0.751–0.786)	0.014
	RF	0.718 (0.706–0.730)	0.677 (0.666–0.688)	0.639 (0.620–0.658)	0.714 (0.698–0.731)	0.017
	CB	0.727 (0.714–0.740)	0.684 (0.671–0.697)	0.648 (0.627–0.669)	0.720 (0.701–0.739)	0.016
2-min	SVM	0.715 (0.703–0.727)	0.668 (0.656–0.680)	0.640 (0.625–0.655)	0.697 (0.680–0.714)	0.029
	KNN	0.700 (0.687–0.713)	0.666 (0.654–0.678)	0.600 (0.582–0.618)	0.732 (0.715–0.749)	0.017
	RF	0.705 (0.694–0.716)	0.672 (0.662–0.682)	0.616 (0.599–0.633)	0.728 (0.710–0.745)	0.017
	CB	0.718 (0.706–0.730)	0.680 (0.668–0.692)	0.622 (0.604–0.640)	0.738 (0.719–0.757)	0.019
1-min	SVM	0.723 (0.709–0.737)	0.661 (0.650–0.672)	0.588 (0.571–0.605)	0.731 (0.711–0.751)	0.018
	KNN	0.699 (0.686–0.712)	0.658 (0.647–0.669)	0.549 (0.529–0.569)	0.763 (0.747–0.779)	0.016
	RF	0.685 (0.671–0.699)	0.643 (0.631–0.655)	0.601 (0.579–0.622)	0.683 (0.661–0.705)	0.033
	CB	0.708 (0.695–0.721)	0.659 (0.648–0.670)	0.608 (0.588–0.628)	0.707 (0.689–0.725)	0.032

*AUC* area under the receiver operating characteristic curve, *CB* CatBoost classifier, *CI* confidence interval, *KNN* k-nearest neighbors, *p* permutation test p-value, *RF* random forest, *SVM* support vector machine.

The overall best performance was achieved by SVM trained on task related HRV data with a time window length of 5 min. The model (AUC = 0.843, accuracy = 74%) that best represented the distribution of SVM classifiers in this particular classification problem used the non-linear features DFA2 and approximate entropy, as well as two frequency-domain indices, VLF and LF/HF ratio, whereas the optimal hyperparameters were 10^1^ for *C* and 10^–2^ for *γ* with the radial basis function. This model had a sensitivity of 72% and a specificity of 76%. Regarding the other main classification problem (i.e., training the models on resting HRV data), the best performance was observed (AUC = 0.751, accuracy = 74%) when we used KNN (*k* = 18) as the classifier with a time window of 4 min (i.e., the longest time window available for the complete dataset in case of training on resting HRV data). This model used the features D2, DFA2, VLF, and SD2. The sensitivity and specificity of the model were 68% and 80%, respectively.

### Regression

**Figure 4:**
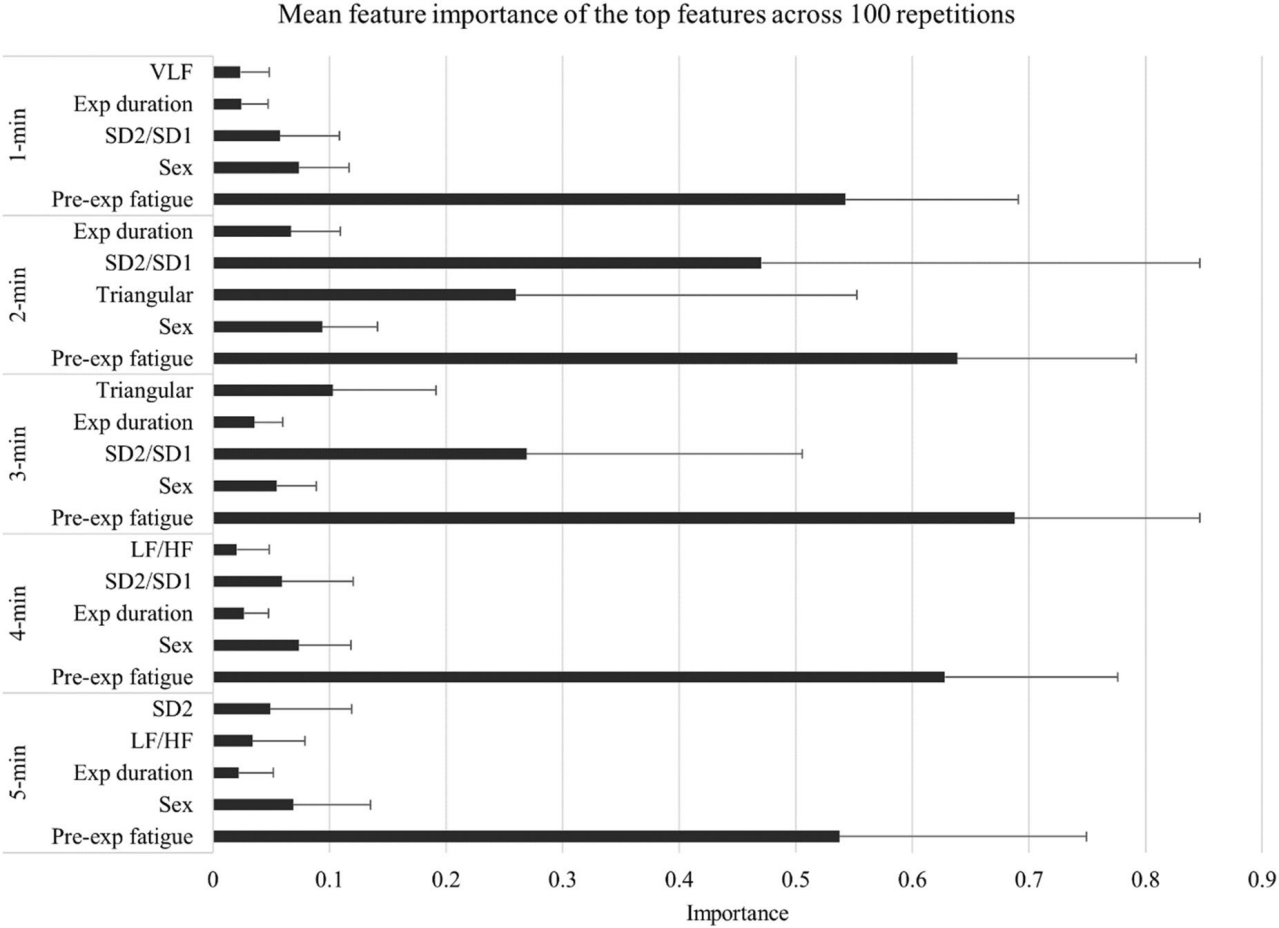
Importance values of the most frequently selected features for regression. Error bars represent 1 standard deviation. *Exp duration* duration of the experiment, *LF/HF* ratio of the low and high-frequency components, *Pre-exp fatigue* the level of subjective fatigue prior to the experiment, *SD1/SD2* ratio of the width and the length of the Poincaré plot, *SD2* the length of the Poincaré plot, *Triangular* HRV triangular index, *VLF* natural logarithm of the very low frequency component.

Regression algorithms were trained to predict the level of subjective fatigue measured after prolonged cognitive task performance. The most frequently selected features for regression modeling are presented in Fig. 4. The results of cross-validation are reported in the Supplementary Materials (see Supplementary Table S2). The performances of the LASSO and elastic net regression models, and CB models are summarised in Table 3. All models differed significantly from the permuted null-distribution, except for the models trained on HRV data calculated for 5-min time window, most probably because of missing data in the 2-back experiment. The CB regressor model trained on 3-min HRV data showed the overall best performance. The model (*R*^2^ = 0.248, RMSE = 17.21, number of estimators = 200, depth = 3, l2 leaf regularisation = 4) that best represents the distribution used the following features: pre-experiment VAS, sex, experiment duration, maximum heart rate, HRV triangular index, SD2, and the SD1/SD2 ratio. The correlation between predicted and true values was moderate (r = 0.628).

**Table 3:** Results of the regression models predicting the level of post-experiment subjective fatigue.

Time window/algorithm		Evaluation metrics (test set)		
		Median R^2^ (Q1–Q3)	Median RMSE (Q1–Q3)	*p*
5-min^a^	Elastic net	0.003 (− 0.261 to 0.202)	18.730 (17.218–21.221)	0.116
	LASSO	− 0.031 (− 0.317 to 0.192)	18.931 (17.289–21.222)	0.292
	CB	0.028 (− 0.218 to 0.168)	19.536 (17.067–21.014)	0.142
4-min	Elastic net	0.224 (− 0.021 to 0.321)	17.114 (15.491–18.323)	0.006
	LASSO	0.205 (− 0.039 to 0.327)	17.092 (15.719–18.415)	0.012
	CB	0.213 (0.024 to 0.324)	17.082 (15.291–18.845)	0.015
3-min	Elastic net	0.209 (0.024 to 0.369)	16.648 (15.505–18.218)	0.006
	LASSO	0.206 (0.015 to 0.368)	16.855 (15.510–18.463)	0.011
	CB	0.248 (0.058 to 0.335)	17.058 (14.940–18.864)	0.008
2-min	Elastic net	0.216 (− 0.028 to 0.380)	16.613 (15.053–18.450)	0.006
	LASSO	0.187 (− 0.049 to 0.364)	16.854 (15.362–18.648)	0.017
	CB	0.196 (0.034 to 0.321)	17.294 (15.500–19.244)	0.016
1-min	Elastic net	0.165 (− 0.038 to 0.308)	17.311 (15.528–19.714)	0.014
	LASSO	0.141 (− 0.053 to 0.315)	17.444 (15.703–19.782)	0.025
	CB	0.195 (− 0.020 to 0.313)	17.407 (15.444–19.252)	0.011

*CB* CatBoost regressor, *LASSO* Least absolute shrinkage and selection operator, *p* Permutation test p-value, *RMSE* Root mean squared error.

^a^Please, note that the 5-min time window could not be used in the 2-back experiment.

Among the linear models, the best performance was achieved by elastic net regression trained on 4-min HRV data. The elastic net regression (*R*^2^ = 0.219, RMSE = 16.231, α = 0.08) model that best represent the distribution used the following features: pre-experiment VAS, sex, the duration of the experiment and two HRV indices: SD1/SD2 ratio and the total power. All predictors were positive, indicating that higher levels of pre-experiment subjective fatigue, HRV and longer experiment duration predicted higher levels of post-experiment subjective fatigue. In addition, female participants were predicted to experience more severe post-experiment subjective fatigue compared with males. The correlation between predicted and true values was moderate (r = 0.559).

## Discussion

The main objective of this study was to determine the extent to which classification models and regression models trained on HRV data can detect a fatigue state and predict the level of subjective fatigue that results from prolonged performances of different cognitively demanding tasks, respectively. By combining the datasets of three different experiments that applied different cognitive tasks, we investigated the predictive power of these models when trained on heterogeneous data in terms of fatigue induction. These tasks had different parameters in terms of stimuli, duration, goals and so on, and the perceived levels of workload also differed. Therefore, we argue that the models trained on the combined dataset of these experiments are robust meaning that they are not limited to special task characteristics and show higher levels of generalisability.

Compared with previous studies that used only a single task for fatigue induction, the fatigue classification algorithms in the present study performed at a similar level^23,29–31^; the AUC scores and balanced accuracies in this study ranged from 0.685 to 0.841 and from 68 to 76%, respectively. The predictive power mainly depended on two factors: (1) the time of ECG recording, and (2) the time window used for HRV calculation. One of our major findings was that the predictive power was higher when the models were trained on task-related data compared with resting HRV data. This unique observation suggests that HRV features extracted from a resting ECG recording might be more prone to individual differences making it more complicated for the algorithms to effectively learn. The comparison of sensitivity and specificity scores indicated that although both metrics were lower for models trained on resting data compared with task-related data, specificity (i.e., the classification accuracy for the “non-fatigue” label) decreased to a greater extent than sensitivity. This might be due to higher individual differences in the pre-experiment resting period that could derive from different levels of pre-task anxiety or differences in mood prior to task performance that can be reflected by cardiac activity^58,59^. Another potential explanation could be that the variance was lower in the task-related dataset obtained at the beginning of task performance (i.e., the data with the “non-fatigue” label) leading to more effective model training and better detection of the non-fatigued state.

This finding of better performance of classification based on task related HRV has important practical implications. When designing monitoring systems that detect fatigue and warn people about their fatigued state in order to avoid accidents, one should consider the advantage of task-related data and build a system that monitors and acts during cognitive activity. A system like this would certainly be beneficial in that it would let people optimise the timing of breaks during task performance because it could warn a person to take a rest when it detects fatigue. However, it is important to note that the observed asset of algorithms trained on task-related data might apply only to HRV and may not necessarily generalise to other physiological measures (e.g., brain activity measured by EEG).

Another important result of the classification analyses is that the predictive power increased as a function of time window length used for HRV. This finding is in line with the findings of previous studies^23,32^, and suggests that HRV indices obtained from longer periods of recording are more reliable and informative for fatigue detection. However, because predictive power was still relatively good (i.e., AUC score = 0.82, accuracy = 74.4%) when a shorter, 2-min time window was used on task-related data, researchers as well as practitioners may consider using a 2-min window when fatigue detection in shorter time periods is required. Although the conventional minimum for HRV calculation is 5 min^60^, recent studies have provided support for the reliability of time windows even shorter than 2 min^61,62^.

In line with the notion that the predictive power depends on the time and length of ECG recording, the best performance was achieved by the SVM classifier trained on task-related HRV data calculated for a 5-min time window. The AUC score of 0.84 produced by this model indicates high efficacy and is comparable to the results of previous studies^30,31^. The good performance could be explained by several factors, such as having a large sample size or using recursive feature elimination for feature selection, which has not been used in previous studies. In addition, the inclusion of non-linear HRV indices is likely to be the source of good performance given the fact that most of the selected features (e.g., entropies, DFA1 and DFA2) were obtained by means of non-linear analyses. This latter explanation is further supported by a study that found an association between non-linear HRV indices and mental workload^63^, a psychological concept strongly related to fatigue^3^. More specifically, theoretical models suggest that the perceived level of mental workload plays a crucial role in the emergence of fatigue. Increasing levels of workload is likely to increase feelings of fatigue especially when the perceived benefits of task performance decrease or remain unchanged^3,4,64^. Beside the high efficacy of models trained on task-related data, the models trained on resting data performed relatively well, too. It is interesting that the best AUC score (0.75) achieved by these models in our study (with the KNN classifier) was almost identical to the AUC score (0.74) of the KNN classifier reported by Huang et al. (2018) who trained the algorithm on resting HRV data. This suggests that an AUC score of ~ 0.75 might be the upper limit of predictive power for models trained on resting HRV.

Another aim of this study was to predict the level of subjective fatigue that emerged during prolonged cognitive activity based on pre-experiment resting HRV and other variables. Consistent with previous studies that have investigated the predictive power of resting HRV on subjective fatigue in healthy individuals^65^ and patients with multiple sclerosis^66^, we found that resting HRV contributed to the prediction of post-experiment subjective fatigue. From a methodological point of view, however, our study differs from these previous studies for four reasons. First, the cognitive tasks used for fatigue induction were variable and thus, we found that the predictive power of resting HRV is not limited to a specific cognitive task but can be generalised to a variety of tasks. Second, we examined several time window lengths, and our findings suggest that shorter periods of ECG recording might also be effective for fatigue prediction. Third and finally, in addition to the time- and frequency-domain HRV indices, we included non-linear HRV indices as potential predictors. This decision is justified because the most frequently selected HRV feature was the SD2/SD1 ratio, a non-linear index, which had relatively high overall importance scores.

In addition to the HRV indices, the regression models included demographic, subjective and other variables (e.g., sleep and task duration) as well. As expected, and in line with previous findings^65^, the most important predictor of post-experiment subjective fatigue was the pre-experiment level of subjective fatigue. This indicates that a person is more likely to experience severe fatigue after task performance if their baseline level of fatigue is higher relative to those who feel more rested before task performance. This also highlights that systems designed for fatigue prevention should take into account the baseline level of fatigue. We also found that sex was a useful predictor and that, compared with male participants, female participants reported higher levels of fatigue after task performance. Sex differences in task-related fatigue have received little attention so far, and the previous studies that have included it found no or only marginal, significant sex effects^65,67^ indicating that—consistent with our results—females tend to rate their level of subjective fatigue higher^68^. Nevertheless, this finding provides support for the notion that sex explains a relevant proportion of the variance in fatigue sensitivity, possibly through complex interactions between sex hormones and the dopaminergic systems^69^. The duration of the experiment was also found to be an important predictor. This is in line with other fatigue experiments,^8,70^ suggesting that the level of subjective fatigue increases in a monotonic way with increasing time spent on a cognitive task.

## Limitations

This study has several limitations. The resting periods in the 2-back experiment were only 4-min long and thus, model training on 5-min resting HRV data was conducted on a smaller dataset. This had the strongest effect in the case of regression because models trained on 5-min HRV data performed remarkably more poorly compared with the other models. In addition, similar to most machine learning studies that aimed at the prediction of fatigue, in this study we investigated only within-site generalisability (i.e., how the models generalise when both the training and testing sets were obtained in the same laboratory) and thus can provide no information about between-site generalisability (i.e., how the models generalise to external data sets obtained by other researchers in different locations). Further studies should be conducted in collaboration with independent researchers from different laboratories to gather an external dataset for model testing in order to assess between-site generalisability.

## Conclusions

This study shows that detecting fatigue on the basis of HRV data via machine learning is effective even if fatigue is induced by different cognitive tasks, leading to more heterogeneous data for both training and testing. This indicates that the models are not strongly influenced by the specific task characteristics which implies higher levels of generalisability. The classifiers trained on ECG data recorded during task performance outperformed those trained on resting data, and we recommend their use in future studies. Finally, regression models predicted the severity of post-experiment subjective fatigue with moderate predictive accuracy. These models included the predictors pre-experiment fatigue, sex, task duration and HRV indices. Similar to classification, longer time windows resulted in better predictions. Both types of machine learning models (i.e., classification and regression models) included HRV indices obtained by non-linear analyses (entropies, Poincaré plot, detrended fluctuation analyses) and these indices were particularly important predictors of fatigue in the machine learning models.

## Supplementary Information

Supplementary Information 1.

## Data Availability

The data and materials for all experiments are available at https://data.mendeley.com/datasets/kbjgw4msv5/draft?a=714e3730-325c-47b3-856a-21453c6ad80f.

## Code availability

http://dx.doi.org/10.17632/kbjgw4msv5.1.
