# Supplementary material for: Generalisable machine learning models trained on heart rate variability data to predict mental fatigue
Source: Sci Rep. 2022 Nov 21;12:20023. doi: 10.1038/s41598-022-24415-y (PMC9681752; doi:10.1038/s41598-022-24415-y)
Supplement: Supplementary file 1 — Supplementary Information 1. [file 41598_2022_24415_MOESM1_ESM.pdf]

Supplementary Materials

Generalisable Machine Learning Models Trained on Heart Rate Variability Data to Predict Mental Fatigue

András Matuz*, Dimitri van der Linden, Gergely Darnai, Árpád Csathó

* Correspondence:

András Matuz, Ph. D.

Department of Behavioural Sciences, Medical School

University of Pécs, Szigeti str. 12, Pécs, 7624, Hungary

e-mail: andras.matuz@aok.pte.hu

Phone: +(36) 72 536 256

Task descriptions

Cross-modal switching task (task switching experiment)

A modified version of the cross-modal switching task developed by Lukas et al. (2014) was used (Matuz et al, 2019). To indicate the relevant modality of the stimulus, each trial started with a visual cue (a white cross, 1.5 cm x 1.5 cm, visual angle of 1.25°) or an auditory cue (600 Hz tone, 45dB). The cues were presented for 200 ms. The number of consecutive trials repeating the same cue modality (repetition trials) varied between 2 and 5. After the cue, visual and auditory stimuli were presented simultaneously with a presentation time of either 100 ms (short) or 300 ms (long). Trials could be congruent if the duration of the auditory and visual stimuli were the same, or incongruent if the duration of the two stimuli differed. The visual stimulus was a white diamond (1.5 cm x 1.5 cm, visual angle of 1.25°) presented centrally, and the auditory stimulus was a 400 Hz tone (45dB). By key press on a response pad, participants were asked to indicate the duration of the cued stimulus (short or long). The stimulus-response mapping was counterbalanced across participants. A trial was terminated when a response was given or after 2500ms. The response-cue interval was constantly 1500ms. We emphasized the equal importance of speed and accuracy to the participants.

Gatekeeper task (2-back experiment)

A modified version of the Gatekeeper task developed by Heathcote et al. (2014, 2015) was used (Matuz et al, 2021). Gatekeeper task is a dual 2-back task with visual and auditory stimuli, and it has a game-like character given by the task instructions. We instructed participants that "they were in a training to become a nightclub doorperson, and that their task was to allow in only cool patrons. A patron tries to gain access through one of the three doors, as indicated by the door being highlighted, and by saying one of the three password letters" (see Heathcote et al., 2015, pp. 976).

On each trial, the visual (i.e. door images) and auditory stimuli (i.e. spoken letters) were presented simultaneously. For the visual stimulus, an image of 3 doors (5.58o x 7.65o visual angle) was shown in the center of the screen; one of the doors was always highlighted by light red color. For the auditory stimulus, one of three vowel letters was spoken by regular speakers (A, E, I; phonetic symbols: ɒ, ɛ, iː). Four different stimulus conditions were prepared: Dual target, Single visual target, Single auditory target, and No target. For the Dual target condition, both the visual and auditory stimuli matched with the stimuli shown two trials earlier (2-back match). For the two single target conditions, the 2-back match occurred in one of the stimulus modalities only: either for the auditory stimulus in the Single auditory target condition) or for the visual stimulus in Single visual target condition. For the No target condition, neither of the stimuli had a two-back match. The 50% of the trials were target trials (i.e. Dual target, Single auditory target, and Single visual target trials). A trial terminated by the response or by the lapse of 2.5s without response, and a new trial began after a 2.5s interval after response.

Participants were needed to indicate by key press on a response pad whether they block (in case of a two-back match in any stimulus modality) or allow the entrance of the patron (no two-back match in the stimulus modalities). The order of the keys was counterbalanced across participants. It was emphasized that both speed and accuracy are equally important.

Semantic Stroop test (Stroop experiment)

In the semantic Stroop test, two modality conditions were introduced. In the auditory condition, participants had to attend the auditory stimulus and ignore the visual stimulus, while in the visual condition, they had to attend the visual stimulus and ignore the auditory one. The modality condition changed after every 12 consecutive trials in an alternating fashion (i.e. after 12 trials the participants always had to attend to other modality). Visual cues

accompanied by an auditory warning signal indicated the modality that had to be attended in the next 12 trials. The visual cue was either the word “Auditory” or “Visual” presented in the center of the screen for 1000 ms. The auditory warning signal was a 800 Hz tone presented for 100 ms with an intensity of approx. 45db. The auditory and the visual stimuli were spoken and written names of animals (birds and mammals), respectively, presented for 700 ms. The two stimuli were presented simultaneously. Participants were asked to judge whether the attended written or spoken name of the animal presented in the actual trial referred to a bird or a mammal. Participants responded in a time window of 1500 ms by pressing one key on the response box for birds or another key for mammals. The intertrial-interval varied between 500 and 3000 ms.

Sleep duration measurement in the experiments

In each experiment, participants were asked to have a decent sleep during the night prior to the experiment. Sleep duration was measured by self-report and by an actigraph (except for the Stroop experiment). The mean duration of sleep prior to the experiments was 7.7 hours (*SD* = 1.56) for the self-reports, while it was 7.79 hours (*SD* = 1.5) based on the actigraph measurement. Thus, the participants were well-rested before the fatigue-induction.

Feature selection for classification models

Feature selection was performed on the training set in three steps. First, the importance of each variable was computed by random forest classifier (number of estimators = 200). Second, for highly correlated features (i.e. a Pearson’s r-value greater than .7), the one with the lower importance was removed. Third and finally, recursive feature elimination with 5-fold cross-validation (5-CV) was applied to select the best set of features. Importantly, this feature selection procedure was performed separately for each classification problem (i.e. training on task-related vs. resting HRV data) and each time window (i.e. 1-5 minutes).

Hyperparamters tuned for classification models

The hyperparameter space of the support vector machine algorithm consisted of linear and radial basis functions for kernel, the set $\{10^0, 10^1, 10^2\}$ for C and the set $\{10^0, 10^{-1}, 10^{-2}\}$ for γ. For the k-nearest neighbors algorithm, k values from 1 to 20 were examined to identify the most optimal one. Finally, for random forest, the optimized parameters were maximum depth (ranging from 3 to 6) and the number of estimators (10, 50, 100 and 200).

Procedure for permutation tests

To conduct permutation tests for the classification models, the procedure described in Boeke et al. (2020) was followed. On each iteration, a model was trained on the training dataset with shuffled class labels (i.e. predictors and class labels were mismatched) and an AUC score was calculated based on the performance of the model on the (unshuffled) testing dataset. We thus generated the null-distribution of AUC scores, and a p-value was obtained by calculating the ratio of the number of cases that resulted in higher AUC scores compared to the actual model and the number of iterations.

For regression, we followed the same procedure described above. On each iteration, the model was trained on the shuffled training dataset (i.e. where the predictors and the outcome variable did not match) and the level of subjective fatigue was predicted in the (unshuffled) testing set. From the observed R^2 values, we generated the null-distribution of R^2 values and a p-value was obtained by calculating the ratio of the number of cases that resulted in R^2 values higher than the actual R^2 and the number of iterations.

Model performances on the training sets

Table S1. Results of the 5-fold cross-validation for classification models trained on resting and task-related HRV. Scores represent the area under the curve with the 95% confidence interval.

**Time window/ Algorithm**		**Time of ECG recording**	
		Resting HRV	Task-related HRV
**5-min**	SVM	.798 (.791 - .805)	.862 (.857 - .867)
	KNN	.795 (.787 - .803)	.846 (.841 - .851)
	RF	.783 (.773 - .793)	.834 (.828 - .840)
	CB	.898 (.891 - .905)	.933 (.927 - .939)
**4-min**	SVM	.789 (.783 - .795)	.853 (.848 - .858)
	KNN	.797 (.790 - .804)	.839 (.834 - .844)
	RF	.771 (.764 - .778)	.837 (.830 - .844)
	CB	.912 (.902 - .922)	.929 (.923 - .935)
**3-min**	SVM	.786 (.779 - .793)	.845 (.840 - .850)
	KNN	.783 (.776 - .79)	.827 (.821 - .833)
	RF	.761 (.753 - .769)	.825 (.819 - .831)
	CB	.903 (.893 - .91	.923 (.916 - .93)
**2-min**	SVM	.766 (.760 - .772)	.845 (.840 - .850)
	KNN	.751 (.744 - .758)	.833 (.828 - .838)
	RF	.753 (.744 - .762)	.831 (.826 - .836)
	CB	.884 (.874 - .894)	.933 (.926 - .94)
**1-min**	SVM	.752 (.744 - .760)	.813 (.807 - .819)
	KNN	.734 (.725 - .743)	.805 (.798 - .812)
	RF	.733 (.725 - .741)	.805 (.799 - .811)
	CB	.899 (.889 - .909)	.918 (.91 - .926)

Abbreviations: CB = CatBoost classifier; KNN = k-nearest neighbors; RF = random forest; SVM = support vector machine

Table S2. Results of the 5-fold cross-validation for regression models.

Time window / algorithm		Evaluation metrics (training set)	
		Median R^2 (Q1 – Q3)	Median RMSE (Q1 – Q3)
**5-min**	*Elastic net*	.493 (.444 - .536)	13.892 (13.183 - 14.711)
	LASSO	.498 (.453 - .545)	13.794 (13.041 - 14.632)
	CB	.807 (.756 - .847)	8.598 (7.444 – 9.795)
**4-min**	*Elastic net*	.479 (.438 - .513)	13.689 (13.416 - 14.147)
	LASSO	.486 (.443 - .518)	13.643 (13.364 - 14.043)
	CB	.774 (.740 - .818)	9.113 (8.300 – 9.554)
**3-min**	*Elastic net*	.515 (.461 - .560)	13.155 (12.808 - 13.699)
	LASSO	.518 (.468 - .561)	13.081 (12.757 - 13.644)
	CB	.788 (.754 - .816)	8.799 (8.151 – 9.516)
**2-min**	*Elastic net*	.565 (.484 - .608)	12.367 (11.993 - 13.518)
	LASSO	.572 (.492 - .610)	12.276 (11.933 - 13.365)
	CB	.788 (.728 - .828)	8.622 (7.923 – 9.784)
**1-min**	*Elastic net*	.467 (.412 - .494)	14.126 (13.488 - 14.601)
	LASSO	.473 (.424 - .501)	14.025 (13.357 - 14.497)
	CB	.772 (.729 - .804)	9.141 (8.475 – 9.812)

Abbreviations: CB = CatBoost regressor; LASSO: least absolute shrinkage and selection operator; RMSE: root mean squared error
